# Prenatal Diagnosis of *MSL2*‐Related Ventriculomegaly in Association With an Inherited 15q13 Microduplication

**DOI:** 10.1111/cge.70071

**Published:** 2025-09-15

**Authors:** Omar Zgheib, Thomas Rio Frio, Jean‐Marie Pellegrinelli, Stefania Gimelli, Caterina Marconi, Delphine Le Mercier, Monica Rebollo Polo, Céline Habre, Joël Fluss, Russia Ha‐Vinh Leuchter, Marc Abramowicz, Rosalinda Giannini, Siv Fokstuen

**Affiliations:** ^1^ Division of Medical Genetics Diagnostics Department, Geneva University Hospitals Geneva Switzerland; ^2^ Molecular Diagnostics Laboratory Diagnostics Department, Geneva University Hospitals Geneva Switzerland; ^3^ Division of Fetal Medicine, Department of Obstetrics, Gynecology and Pediatrics Geneva University Hospitals Geneva Switzerland; ^4^ Cytogenetics Laboratory Diagnostics Department, Geneva University Hospitals Geneva Switzerland; ^5^ Division of Radiology Diagnostics Department, Geneva University Hospitals Geneva Switzerland; ^6^ Division of Neuropediatrics, Department of Obstetrics, Gynecology and Pediatrics Geneva University Hospitals Geneva Switzerland; ^7^ Division of Child Development, Department of Obstetrics, Gynecology and Pediatrics Geneva University Hospitals Geneva Switzerland

## Abstract

The Male‐Specific Lethal 2 Homolog (*MSL2*) gene was recently reported to be responsible for a novel, rather severe neurodevelopmental syndrome including brain abnormalities. We report the first prenatal case of an MSL2‐related pathology caused by a *de novo MSL2* splice variant (c.142+1G>T). RNA study on amniotic fluid cells showed an intronic inclusion and frameshift, consistent with loss‐of‐function intolerance. The fetus, who presented with bilateral moderate ventriculomegaly, also carried a paternally inherited 15q13 microduplication. Brain MRI at 2 and 4 months of age showed stable, mildly enlarged lateral ventricles. Clinical evaluation at 11 months revealed only a mild developmental delay. This case illustrates the challenges in predicting the postnatal outcome of recently characterized syndromes with limited documented cases, especially in association with a second independent genetic anomaly. Follow‐up will be crucial to better define the developmental impact of this first reported *MSL2* splice mutation in combination with the 15q13 microduplication, and characterization of more patients with *MSL2* mutations will contribute to expanding the phenotypic spectrum.

## Graphical abstract

We hereby report the first prenatal diagnosis of MSL2‐related pathology, namely ventriculomegaly. Favorable 11‐month follow‐up illustrates the challenges in predicting postnatal outcomes for genetic anomalies linked to recently characterized phenotypes with limited documented cases. More patient characterization will contribute to better understanding of penetrance, phenotypic diversity, and eventual genotype–phenotype correlation.

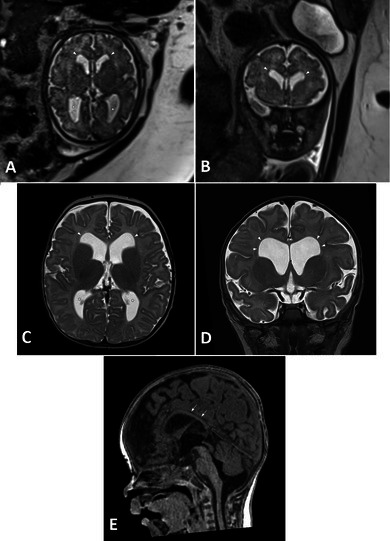

## 1 Introduction

The Male‐Specific Lethal 2 Homolog (*MSL2*) gene (OMIM 614802) was recently reported to be responsible for a novel neurodevelopmental syndrome of variable severity (Karayol‐Borroto‐Haghshenas neurodevelopmental syndrome, OMIM 620985). *MSL2* codes for a subunit of the MSL complex, an important epigenetic regulator of gene expression.

MSL2 is thought to ensure biallelic gene expression in mammals, and reported variants lead to a specific blood DNA methylation signature, but the exact molecular mechanism underlying the disorder is not yet fully understood [1, 2]. Further studies are warranted to better understand MSL2 function and dysfunction in chromatin remodeling, gene expression, and downstream developmental processes.

Two reports published in 2024 described a total of 28 patients aged between 4 months and 37 years, all showing mild to severe developmental delay, the vast majority with motor delay (88%), speech delay (78%), or hypotonia (75%) [1, 3]. More than a third of patients developed epilepsy, and around half (9/20) had non‐specific abnormalities on postnatal brain MRI, including enlarged ventricles, delayed myelination, white matter loss, or other abnormalities. Behavioral disorders, such as autism spectrum disorder, sleep disorders, dysmorphic features, and visual problems were observed in some patients.

All variants in both publications were reported to be *de novo*, except for three for which parental segregation was not performed and one which was inherited from the mother, who herself had intellectual deficiency. Most variants were nonsense or frameshift, with only two missense variants reported, arguing for haploinsufficiency and loss‐of‐function intolerance as the underlying pathogenic mechanism for the *MSL2*‐related disorder (pLI and LOEUF scores at 1 and 0.17, respectively in gnomAD v.4.0.0).

## 2 Case Description and Methods

We hereby report the first prenatal diagnosis of a *MSL2*‐related pathology detected in the context of dilated lateral ventricles (11 mm bilaterally) on fetal ultrasound at 26 weeks of gestational age (GA). This was the first pregnancy of a non‐consanguineous couple of European descent with unremarkable family history. First‐trimester screening revealed a low risk for trisomies 13, 18, and 21, and fetal ultrasound was within the normal range until 26 GA, when bilateral ventriculomegaly was detected. Thereupon, amniocentesis was performed at 26 1/7 GA. Quantitative fluorescent PCR analysis showed no aneuploidy for chromosomes 13, 18, 21, or the gonosomes. In order to conclude rapidly about *de novo* or inherited copy number variants, we subsequently performed trio microarray analysis, which revealed a paternally inherited microduplication on the long arm of chromosome 15, located in the 15q13.2q13.3 region (gain of 1.97 Mb). This gain included 7 OMIM genes, notably cholinergic receptor neuronal nicotinic alpha polypeptide A (*CHRNA7*) and OTU domain‐containing protein 7A (*OTUD7A*), and corresponded to the recurrent 15q13.2–q13.3 microduplication associated with neurodevelopmental disorders of incomplete penetrance and variable expressivity. In fact, the fetus' father was known for minor learning difficulties and a small head circumference (52.3 cm, 3rd percentile). Fetal karyotype was normal (46,XY). As the 15q13 microduplication was not known to be associated with ventriculomegaly, the parents agreed to go on with a prenatal trio clinical exome analysis including 4, 486 Mendelian genes known to be associated with disease with a high level of evidence. Our targeted exome analysis revealed the following *de novo* splice heterozygous variant in the *MSL2* gene: c.142+1G>T (reference sequence NM_018133). Mutations in this gene had just been reported to cause a neurodevelopmental syndrome [1, 3].

**FIGURE 1:**
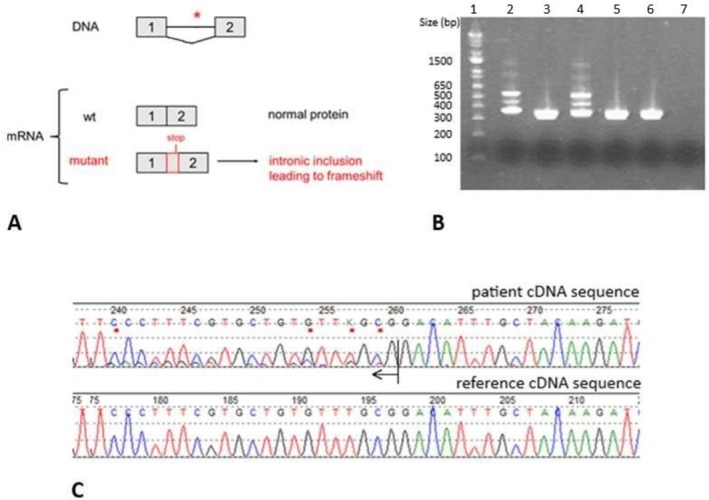
RNA study. (A) Transcription product of wild‐type (wt) and mutant *MSL2*. (B) Agarose gel electrophoresis showing the migration pattern of cDNA products after reverse transcription of mRNA and amplification with exon1 (forward) and exon2 (reverse) primers. Lane 1: Invitrogen 1 Kb Plus DNA Ladder; Lane 2: patient amniocyte cDNA; Lane 3: control amniocyte cDNA; Lane 4: patient amniocyte DNase‐treated cDNA; Lane 5: control amniocyte DNase‐treated cDNA; Lane 6: blood control; Lane 7: negative control. The high‐intensity bands in lanes 2–6 correspond to the wt mRNA product; the weak‐intensity bands in lanes 2 and 4 migrating between 400 and 500 bp correspond to the mRNA product with the inclusion of the first 88 bases of intron 1. The medium‐intensity bands in lanes 2 and 4 migrating between 500 and 650 bp correspond to the heteroduplex of wt cDNA and cDNA with the intronic inclusion. (C) Chromatogram showing in the upper panel the reverse cDNA sequence corresponding to patient amniocyte‐extracted mRNA (presence of intron 1 starting at position 260, corresponding to base 88 in intron 1, indicated by the arrow); reference cDNA sequence in the lower panel.

This variant is absent in the general population (gnomAD v4.1) and in gene mutation databases (HGMD, LOVD, ClinVar). It is predicted to affect the canonical splice donor site in exon 1. We therefore carried out prenatal functional studies on RNA extracted from amniotic fluid, which demonstrated a specific insertion into the messenger RNA of the first 88 bases of intron 1 (ttgagacgctttttattgttcccttttcaatgcttgtttttaaaagccttctgggcagtggtaacgtgagcgggttgacggcctggtg), resulting in the utilization of a downstream splice site and leading to a frameshift and a premature stop codon. This would result in the production of a non‐functional truncated protein: c.142+1G>T, r.142_143ins[u;142+2_142+88], p.(Gly48Valfs*53) (Figure 1).

We therefore classified this *MSL2* variant as likely pathogenic (class 4) according to the American College of Medical Genetics and Genomics (ACMG) variant classification criteria [4]. The following three criteria were taken into account: PS2_moderate (de novo), PM2 (rare), and PVS1_strong (frameshift mutation yielding a truncated protein).

When returning the molecular result to the couple, we emphasized the wide spectrum of clinical manifestations associated with known mutations in *MSL2*, the small number of patients reported to date, as well as the impossibility to predict the individual postnatal clinical outcome of the developing child. After careful consideration, the parents, who had already accepted the uncertainty related to the 15q13.2–q13.3 microduplication, decided to carry out the pregnancy with continuous monitoring.

**FIGURE 2:**
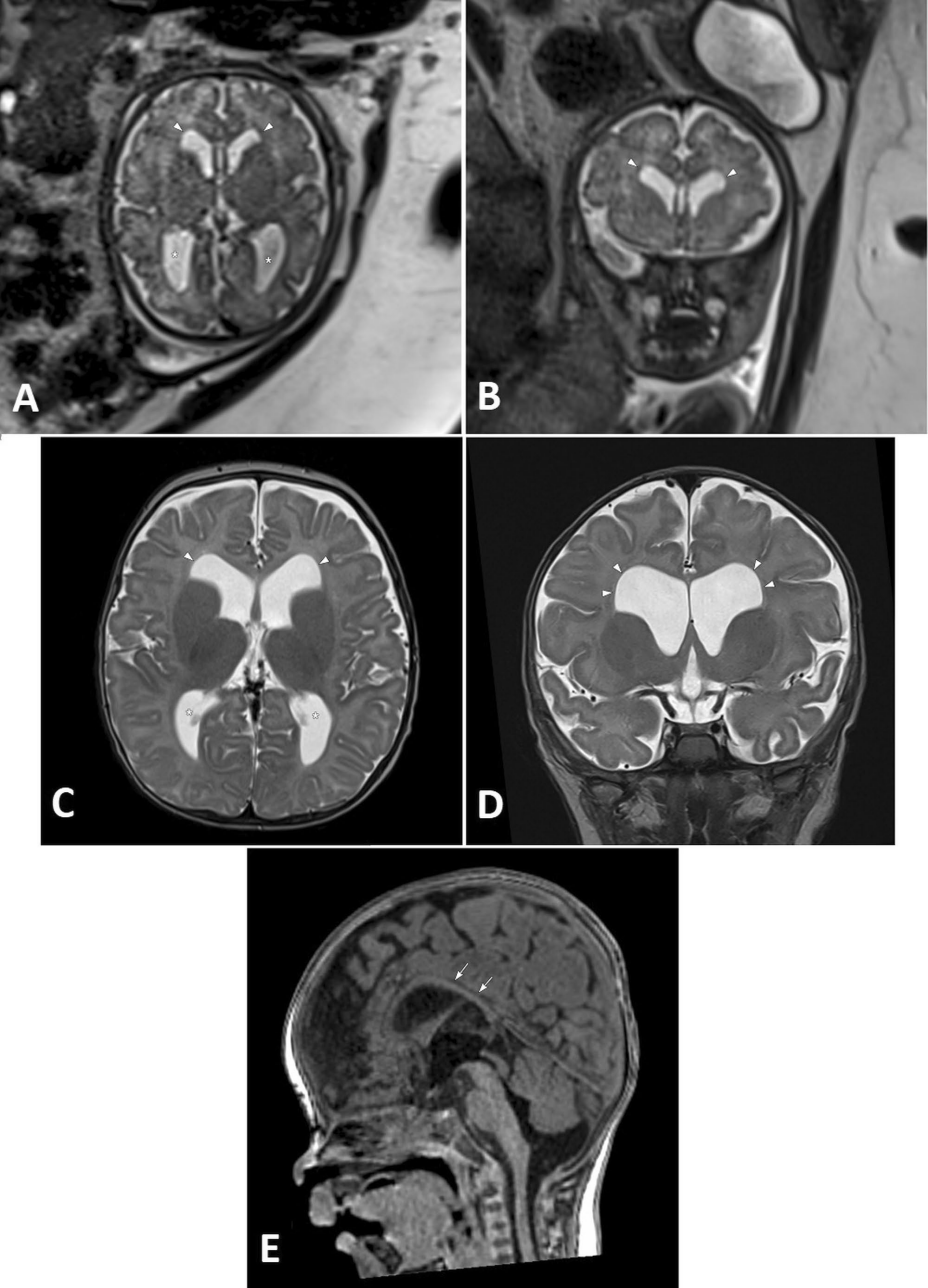
Axial (A) and coronal (B) T2‐weighted HASTE fetal brain MRI at 35 weeks GA: Bilateral and symmetric mild dilatation of the lateral ventricles (white asterisks) and square‐shaped frontal horns (white arrowheads). Axial (C) and coronal (D) T2‐weighted spin‐echo brain MRI at 2 months: Mild bilateral dilatation of the lateral ventricles (white asterisks) with preferential anterior enlargement and square‐shaped frontal horns (white arrowheads). Sagittal reformat 3D T1‐weighted MPRAGE sequence (E): Thin corpus callosum (white arrows).

Fetal brain MRI performed at 29 and 35 weeks GA demonstrated mild bilateral ventriculomegaly (12 mm) with square‐shaped frontal horns and no other structural abnormality, stable over time (Figure 2A,B).

**FIGURE 3:**
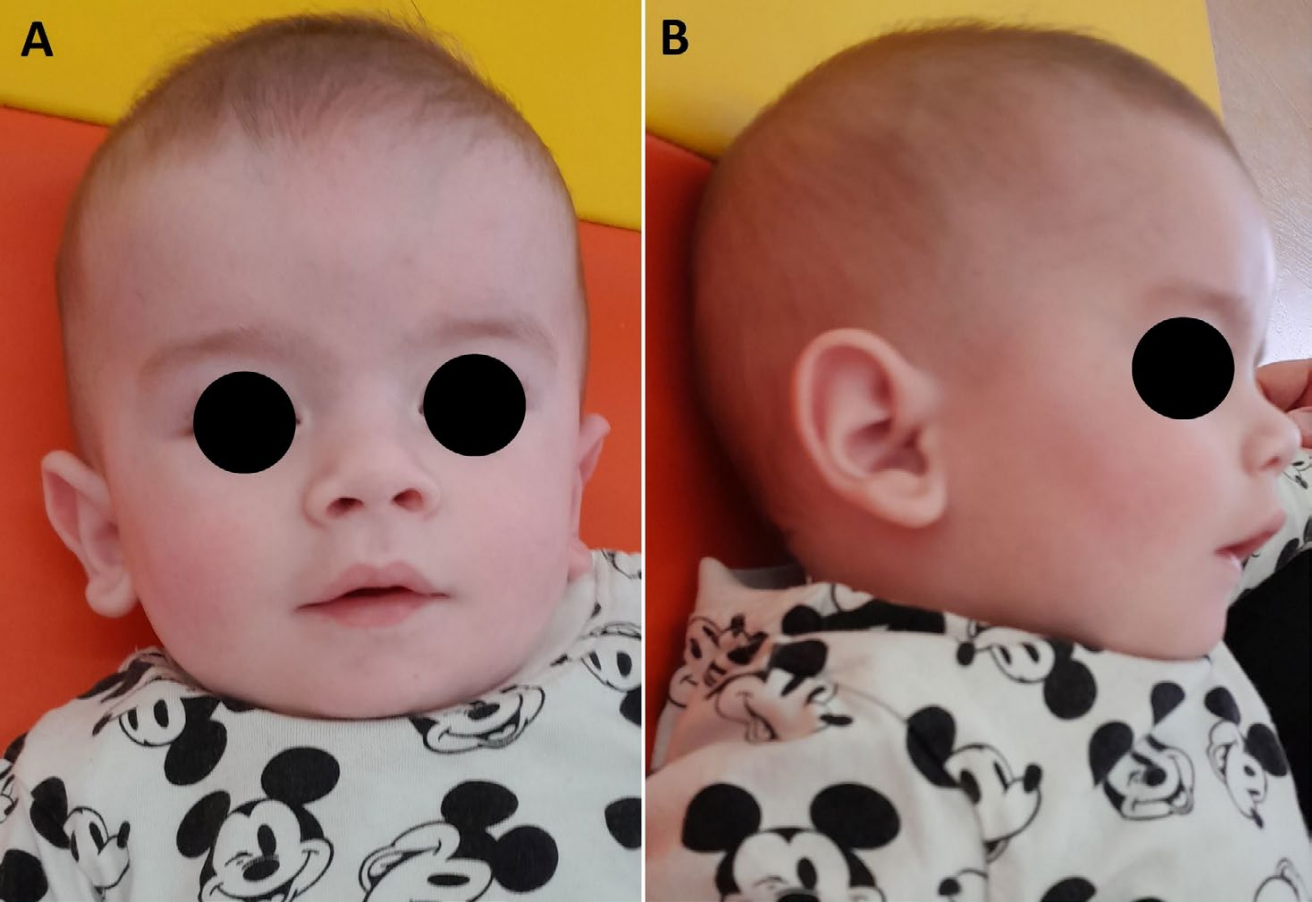
Portrait (A) and profile (B) photos taken at 6 months of age.

The newborn boy was delivered spontaneously at 39 1/7 weeks GA. Birth weight was 2.56 kg (1st percentile), height 50 cm (19th percentile), and head circumference 35 cm (27th percentile). Overall neonatal adaptation was good and neurological examination was within the normal range. A prominent philtrum and *chapeau de gendarme* aspect of the mouth were observed, which the mother also had (Figure 3). Brain ultrasound at 2 days of age showed mildly dilated ventricles (10 mm each). At brain MRI performed at 2 and 4 months, the lateral ventricles were still mildly enlarged at the level of their atria and demonstrated preferential anterior dilatation associated with a square shape of both frontal horns (Figure 2C,D). The dilatation was stable over time, as evidenced by the calculated Evans ratio (0.45) at antenatal and follow‐up MRI. An additional and presumably relevant imaging finding was a thin corpus callosum (Figure 2E) which had been underestimated at fetal MRI.

Clinical evaluation at 11 months of age showed good overall progress with only a mild delay corresponding to a developmental age between 7 and 9 months in the different domains (motor, cognitive, and language/communication), according to the Bayley‐III scales. Head circumference (46.5 cm; 70th percentile), height (74 cm; 50th percentile), and weight (9.3 kg; 50th percentile) were harmonious. Hearing and vision were normal according to age.

## 3 Discussion

To our knowledge, this is the first case of a *MSL2*‐related ventriculomegaly diagnosed prenatally; it is also the first report of a splice variant; all pathogenic or likely pathogenic *MSL2* variants reported so far were nonsense, frameshift, or missense variants.

We characterized the variant prenatally through an RNA study and showed that it would lead to a non‐functional truncated protein, compatible with the proposed loss‐of‐function intolerance for *MSL2*. Given the hitherto rather favorable development of our patient, this case illustrates the difficulty in predicting postnatal outcomes based only on prenatal findings, especially in the presence of likely pathogenic or pathogenic variants in genes associated with recently characterized phenotypes and few reported patients. Characterization of more patients with *MSL2* anomalies will contribute to a better understanding of penetrance, expressivity, and genotype–phenotype correlations associated with this newly identified disorder. In this particular case, the phenotype may be further complicated by the 15q microduplication, itself known to be of incomplete penetrance and variable expressivity.

In summary, we identified, in the context of prenatal bilateral ventriculomegaly, two independent genetic anomalies: an inherited chromosomal anomaly of incomplete penetrance and variable expressivity but not known to be linked to ventriculomegaly, and a novel *de novo* splice *MSL2* variant that very likely explains the finding. Although both anomalies are known to cause neurodevelopmental disorders, ventriculomegaly has so far only been associated with *MSL2* variants. These two distinct genetic anomalies illustrate the huge challenge of prenatal genetic counseling. *MSL2* variants have so far always been associated with neurodevelopmental delay, albeit of variable severity. The child's rather favorable evolution at 11 months is encouraging, although much too early to conclude about his final development. Clinical and radiological follow‐up will be crucial to better define the impact of the *MSL2* variant and the 15q microduplication on our patient's development. This case also illustrates the importance of continuing to investigate additional genetic causes when the initial genetic results do not allow us to explain the phenotype.

## Author Contributions

O.Z. and S.F. wrote the manuscript. T.R.F. conducted the RNA study on cells from amniotic fluid and prepared Figure 1. O.Z., S.F., J.M.P., D.L.M., J.F., R.G., and R.H.V.L. followed the case clinically. C.H. and M.R.P. interpreted the MRI images. S.G., M.C., T.R.F., M.A. performed and interpreted cytogenetic and molecular analysis. All authors contributed to the manuscript elaboration and reviewed it critically. S.F. supervised the manuscript.

## Ethics Statement

As this study is based on a case report with informed patient consent, formal ethical approval was not required under institutional guidelines.

## Consent

Consent for publication was obtained from the patient's mother and is available upon request.

## Conflicts of Interest

The authors declare no conflicts of interest.

## Data Availability

Data sharing not applicable to this article as no datasets were generated or analysed during the current study.
